# Transcription Factor SiDi19-3 Enhances Salt Tolerance of Foxtail Millet and *Arabidopsis*

**DOI:** 10.3390/ijms24032592

**Published:** 2023-01-30

**Authors:** Shenghui Xiao, Yiman Wan, Shiming Guo, Jiayin Fan, Qing Lin, Chengchao Zheng, Changai Wu

**Affiliations:** State Key Laboratory of Crop Biology, College of Life Sciences, Shandong Agricultural University, Tai’an 271018, China

**Keywords:** *CBLs*, *Di19-3*, *NHXs*, foxtail millet, salt tolerance, *SOSs*

## Abstract

Salt stress is an important limiting factor of crop production. Foxtail millet (*Setaria italica* L.) is an important model crop for studying tolerance to various abiotic stressors. Therefore, examining the response of foxtail millet to salt stress at the molecular level is critical. Herein, we discovered that SiDi19-3 interacts with SiPLATZ12 to control salt tolerance in transgenic *Arabidopsis* and foxtail millet seedlings. *SiDi19-3* overexpression increased the transcript levels of most Na^+^/H^+^ antiporter (*NHX*), salt overly sensitive (*SOS*), and calcineurin B-like protein (*CBL*) genes and improved the salt tolerance of foxtail millet and *Arabidopsis*. Six *SiDi19* genes were isolated from foxtail millet. Compared with roots, stems, and leaves, panicles and seeds had higher transcript levels of *SiDi19* genes. All of them responded to salt, alkaline, polyethylene glycol, and/or abscisic acid treatments with enhanced expression levels. These findings indicate that *SiDi19-3* and other *SiDi19* members regulate salt tolerance and other abiotic stress response in foxtail millet.

## 1. Introduction

Salt stress affects physiological factors such as plant growth, development, and yield [1,2,3]. Plants acquire various molecular, biochemical, and physiological responses to tolerate salt stress [1]. Re-establishing ionic equilibrium to enhance salt tolerance is a crucial process for plants [4]. Additionally, the salt overly sensitive (SOS) pathway plays an important role in the response to salt stress in plants. The plasma membrane-localized Na^+^/H^+^ antiporter SOS1 and its regulatory proteins, including SOS2 (a protein kinase, also known as CIPK24), SOS3/calcineurin B-like protein 4 (CBL4), and other CBL proteins, maintain this process [5,6,7,8]. Under salt stress, SOS3 and CBL10 collaborate in the roots and shoots to detect the rise in cytoplasmic calcium and transmit this signal to the downstream serine/threonine protein kinase SOS2. Subsequently, SOS2 phosphorylates SOS1 to enhance Na^+^/H^+^ exchange activity and improve plant salt tolerance [1,3]. Simultaneously, tonoplast-localized Na^+^/H^+^ antiporters (NHXs) that sequester Na^+^ within vacuoles also play vital roles in re-establishing ionic homeostasis under salt stress [9,10]. Loss of function of *SOS* and *NHX* genes results in hypersensitivity to salt stress owing to high Na^+^ accumulation in the cytosol. In contrast, overexpression of the products of these genes improves salt tolerance owing to the low Na^+^ accumulation in the cytosol.

The Cys2/His2-type zinc-finger protein motif was first identified in transcription factor IIIA from Xenopus laevis which can be induced by drought stress [11,12,13]. Proteins containing the Cys2/His2-type zinc-finger are one of the best-characterized DNA-binding transcription factors in eukaryotes [14]. Di19 (drought induced) protein was firstly isolated from drought treated *Arabidopsis* roots [15]. All seven of the AtDi19 proteins contain two atypical Cys2/His2-type zinc-finger motifs [13]. Every member of the *AtDi19* family, except *AtDi19-2,* has putative nuclear localization signals (NLS), and all but two are localized in the nucleus [13]. Dehydration quickly stimulates the expression of *AtDi19-1* and *AtDi19-3;* however, high-salt stress induces the expression of *AtDi19-2* and *AtDi19-4* [13], indicating the different roles of AtDi19 members in abiotic stress responses. By binding to the TACA(A/G)T element in the promoters of pathogenesis-related 1 (*PR1*), *PR2*, and *PR5* genes, AtDi19-1 contributes to the response of plants to drought stress [14]. Transgenic *Arabidopsis* plants are more sensitive to salt, drought, oxidative, and abscisic acid (ABA) stressors when *GmDi19-5* is overexpressed [16]. Additionally, *GhDi19-1* and *GhDi19-2* are implicated in ABA signal transduction and plant responses to salt and drought stresses [17]. *ZmDi19*-1 overexpression enhances salt tolerance in *Arabidopsis* by affecting the expression of stress-related genes [18]. The importance of *TaDi19A* in abiotic stress has been discussed, along with some potential modes of action [19]. By directly interacting with the promoter of *DREB2A* in rice, *OsDi19-4* acts downstream of OsCDPK14 to positively control ABA response [20]. Furthermore, PheDi19-8 directly binds to the promoter of *DREB2A*, and *PheCDPK22* overexpression increases *Arabidopsis* sensitivity to drought stress [21]. Overexpression of *PtDi19-2* and *PtDi19-7* increased ABA sensitivity and drought tolerance in *Arabidopsis* [22]. *PtDi19-2* and *PtDi19-7* probably influence the ability of transgenic plants to withstand drought via ABA-dependent signaling pathways [22]. Therefore, Di19 proteins play a crucial role in the responses of various plant species to abiotic stressors. However, whether Di19 proteins control the salt stress response by regulating the SOS pathway remains unclear.

An earliest cultivated cereal crop, foxtail millet, is mostly farmed in northern China and other parts of East Asia. This is a ubiquitous crop growing in various environments [23,24,25,26]. However, the response of foxtail millet plants to salt stress remains unclear. We previously discovered that AtPLATZ2 interacts with AtDi19-3 in yeast two-hybrid tests and adversely affects the salt tolerance of *Arabidopsis* by suppressing the expression of *CBL4/SOS3* and *CBL10/SCaBP8* [27], thereby indicating the role of AtDi19-3 in salt stress responses in *Arabidopsis*. Similarly, we discovered that *SiPLATZ12* from foxtail millet inhibits the expression of the majority of *SOS*, *CBL*, and *NHX* genes, thereby adversely affecting salt tolerance [28]. However, whether SiPLATZ12 interacts with SiDi19-3 to regulate salt tolerance of foxtail millet is unclear. In this study, we verified the interaction of SiDi19-3 with SiPLATZ12 and the function of SiDi19-3 in the salt stress response in foxtail millet and *Arabidopsis*. Furthermore, the effects of SiDi19-3 on the expression of *SiNHXs/AtNHXs*, *SiSOSs/AtSOSs*, and *SiCBLs/AtCBLs* were investigated in transgenic foxtail millet and *Arabidopsis*. The *SiDi19* family was also isolated, and their expression in specific tissues and in response to specific stimuli was determined. Our findings revealed a new positive regulator of the majority of *SOS*, *CBL*, and *NHX* genes that improve plant salt tolerance.

## 2. Results

### 2.1. SiDi19-3 Interacts with SiPLATZ12

**Figure 1:**
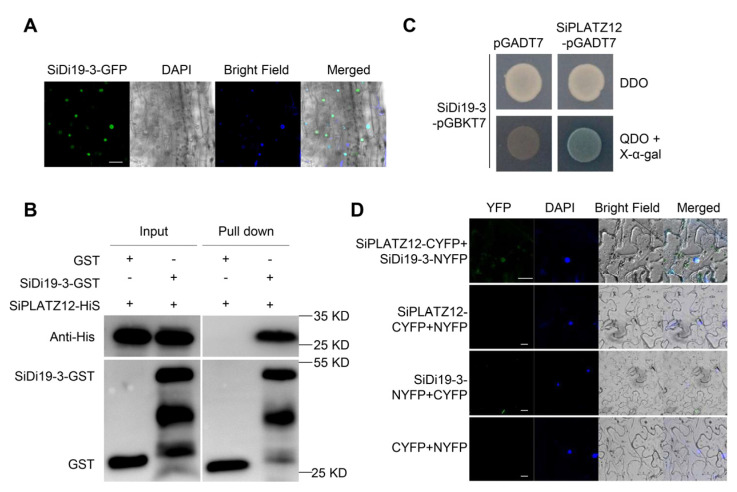
SiDi19-3 interacts with SiPLATZ12. (**A**) Subcellular localization of SiDi19-3 in transgenic hairy-roots of foxtail millet. The photographs were taken using the green channel (GFP fluorescence), blue channel (DAPI fluorescence), bright channel, and their combination under a confocal microscope. Scale bar = 20 μm. (**B**) The SiDi19-3 and SiPLATZ12 interaction tested using yeast two-hybrid assay. DDO, Synthetic Dropout/-Leu-Trp; QDO, Synthetic Dropout/-Leu-Trp-His-Ade. (**C**) The SiDi19-3 and SiPLATZ12 interaction using in vitro pull-down assays. SiPLATZ12-His was incubated with GST or GST-SiDi19-3 purified from *E.coli* and was washed to remove unbound proteins. The bound proteins were eluted and analyzed using immunoblotting with anti-GST and anti-His antibodies. (**D**) The SiDi19-3 and SiPLATZ12 interaction in *N. benthamiana* indicated in bimolecular fluorescence complementation (BiFC) assay. DAPI, nuclear dye, 4′,6-diamidino-2-phenylindole. The photographs were taken using the green channel (YFP fluorescence), blue channel (DAPI fluorescence), bright channel, and their combination under a confocal microscope. Scale bars = 20 μm.

First, we created SiDi19-3-green fluorescence protein (GFP) transgenic hairy roots of foxtail millet. The GFP signal was particularly observed to be merged with the 4′,6-diamidino-2-phenylindole (DAPI) dye, indicating the nuclear localization of SiDi19-3 in the transgenic hairy roots. We then examined whether SiDi19-3 and SiPLATZ12 interacted. Yeast cells co-transformed with the SiPLATZ12-AD and SiDi19-3-BD constructs were able to grow in a synthetic medium deficient in Trp, Leu, His, and 25 mM AbA (aureobasidin A) (quadruple dropout [QDO]), indicating the physical interaction between SiPLATZ12 and SiDi19-3 in yeast cells (Figure 1B). In the pull-down assays, SiPLATZ12-His could be pulled down by SiDi19-3-GST but not by GST alone (Figure 1C). Bimolecular fluorescence complementation (BiFC) assays were carried out. The YFP signal was observed using a confocal microscope when SiPLATZ12 fused to C-terminus of YFP (SiPLATZ12-CYFP) and SiDi19-3 fused to N-terminus of YFP (SiDi19-3-NYFP) were co-transformed into tobacco epidermis cells; however, no YFP signals were observed when SiPLATZ12-CYFP and NYFP, SiDi19-3-NYFP, and CYFP, or NYFP and CYFP were co-transformed (Figure 1D). The YFP signal could also be merged with the DAPI dye. These findings indicate an interaction between SiDi19-3 and SiPLATZ12.

### 2.2. SiDi19-3 Enhances Salt Tolerance of Foxtail Millet

**Figure 2:**
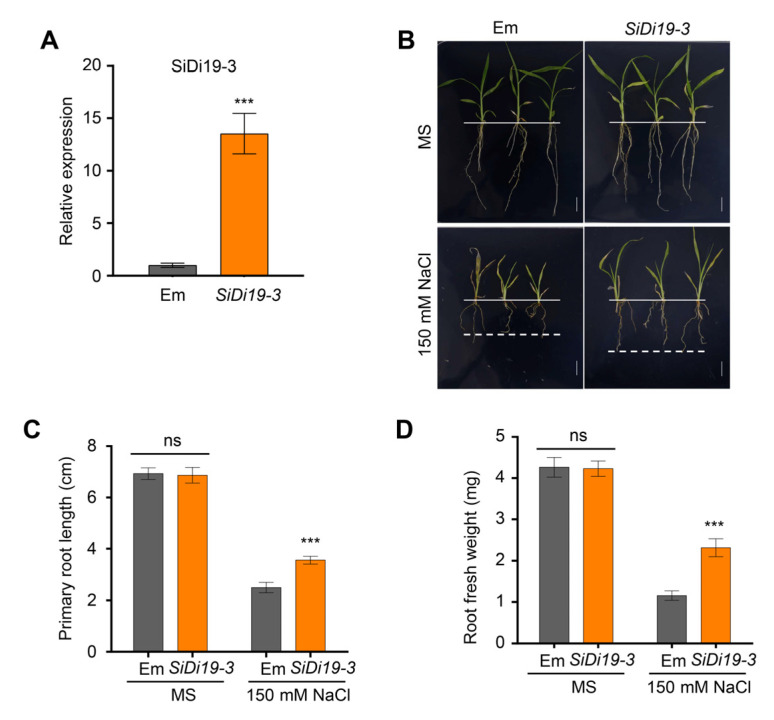
Overexpression of *SiDi19-3* enhances salt tolerance of foxtail millet seedlings. (**A**) Relative expression of *SiDi19-3* in *35S::SiDi9-3* transgenic hairy-roots of foxtail millet seedlings. The empty vector (Em) was used as a control. *SiACTIN7* and *18S rRNA* were used as two normalizing expression values within the calculation leading to the relative expression values. Data shown are means of three biological replicates. At least 30 seedlings were used for each replicate. Data represent the mean ± SEM of three biological repeats. Student’s *t*-test indicated the significance at *** *p* < 0.001 levels. (**B**) Phenotypes of *35S::SiDi9-3* and Em transgenic foxtail millet seedlings with or without salt stress. Scale bars = 1 cm. (**C**) Primary root length and (**D**) root fresh weight of foxtail millet seedlings grown under salt stress or in control conditions. Data represent the mean ± SEM of three biological repeats. Student′s *t*-test indicated significance at *** *p* < 0.001 level. ns, no significance.

To analyze the role of *SiDi9-3* under salt stress, we overexpressed *SiDi19-3* in the hairy roots of ‘Yugu1’ seedlings using K599 agrobacterium-mediated transformation. Notably, the *SiDi19-3* transcript levels in transgenic foxtail millet seedlings were higher than those in the control (Em, empty vector transgenic seedlings) (Figure 2A). The *35S::SiDi19-3* and Em transgenic seedlings were grown in the same way under typical conditions. In *35S::SiDi19-3* transgenic seedlings under salt stress, primary root length was significantly greater and root fresh weight was higher than those in control seedlings (Figure 2B–D), demonstrating that SiDi19-3 positively controls the salt tolerance of foxtail millet seedlings.

### 2.3. SiDi19-3 Increases the Expression of SiSOS, SiCBL, and SiNHX Genes

**Figure 3:**
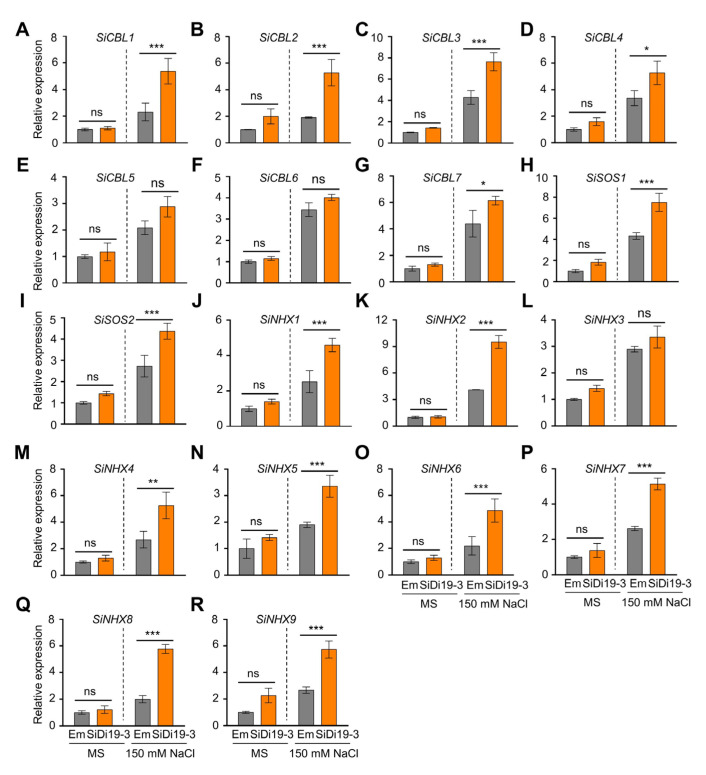
SiDi19-3 upregulates the expression of salt tolerance related genes in foxtail millet. Relative expression levels of *SiCBL1-7* (**A**–**G**), *SiSOS1-2* (**H**,**I**), and *SiNHX1-9* (**J**–**R**) in transgenic foxtail millet hairy roots treated with 150 mM NaCl for 24 h or not. *SiACTIN7* and *18S rRNA* were used as two normalizing expression values within the calculation leading to the relative expression values. Data shown are means of three biological replicates. Data represent the mean ± SEM of three biological repeats. Student′s *t*-test showed the significance at * *p* < 0.05, ** *p* < 0.01, and *** *p* < 0.001 levels. ns, no significance.

To better understand how SiDi19-3 controls the salt stress response in foxtail millet seedlings, we evaluated the transcript levels of salt tolerance related genes, including *SiSOSs*, *SiCBLs*, and *SiNHXs* (Figure 3). Under salt stress, compared to the Em transgenic foxtail millet seedlings, *SiDi19-3*-overexpression seedlings raised the transcript levels of all *SiCBLs,* except *SiCBL5* and *SiCBL6;* all *SiNHXs*, except *SiNHX3*; *SiSOS1*; and *SiSOS2.* However, under normal conditions, *SiDi19-3* overexpression did not alter the transcript levels of them. These findings implied that the majority of the analyzed *NHX*, *SOS*, and *CBL* genes were upregulated by SiDi19-3 under salt stress.

### 2.4. Heterologous Expression of SiDi19-3 Increases Salt Tolerance of Arabidopsis

**Figure 4:**
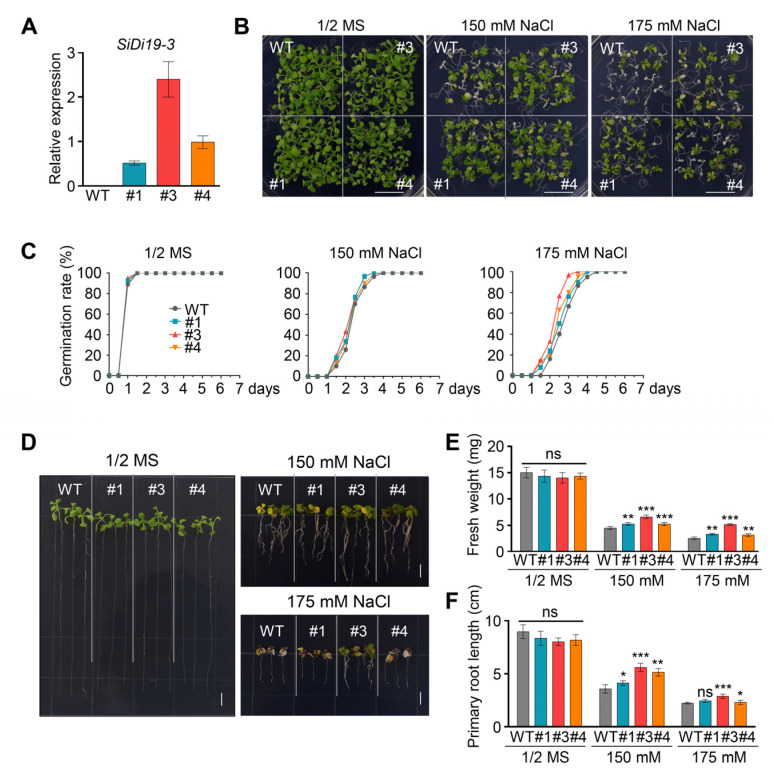
Overexpression of *SiDi19-3* enhances salt tolerance of transgenic *Arabidopsis*. (**A**) *SiDi19-3* transcript levels in wild type (WT) and *SiDi19-3* transgenic *Arabidopsis*. (**B**,**C**) Phenotypes and germination rate of WT and *SiDi19-3* transgenic *Arabidopsis* seeds cultured on half–strength (1/2) MS medium containing 150 mM and 175 mM NaCl or in control conditions. At least 50 seeds were used for each replicate. Three biological repeats were performed. Scale bars = 1 cm. (**D**) Phenotypes of 3-day-old uniformly developed seedlings of WT and three *SiDi19-3* transgenic *Arabidopsis* seedlings grown on 1/2 MS medium with or without 150 mM and 175 mM NaCl for 14 days. Scale bars = 1 cm. (**E**,**F**) Fresh weight and primary root length of WT and SiDi19-3 transgenic *Arabidopsis* seedlings treated as described in (**D**). At least 30 seedlings were used for each replicate. Data shown are means of three biological replicates. Data represent means ± SEM of three replicates. Student’s *t*-test indicated the significance at * *p* < 0.05, ** *p* < 0.01, and *** *p* < 0.001 levels. ns, no significance.

**Figure 5:**
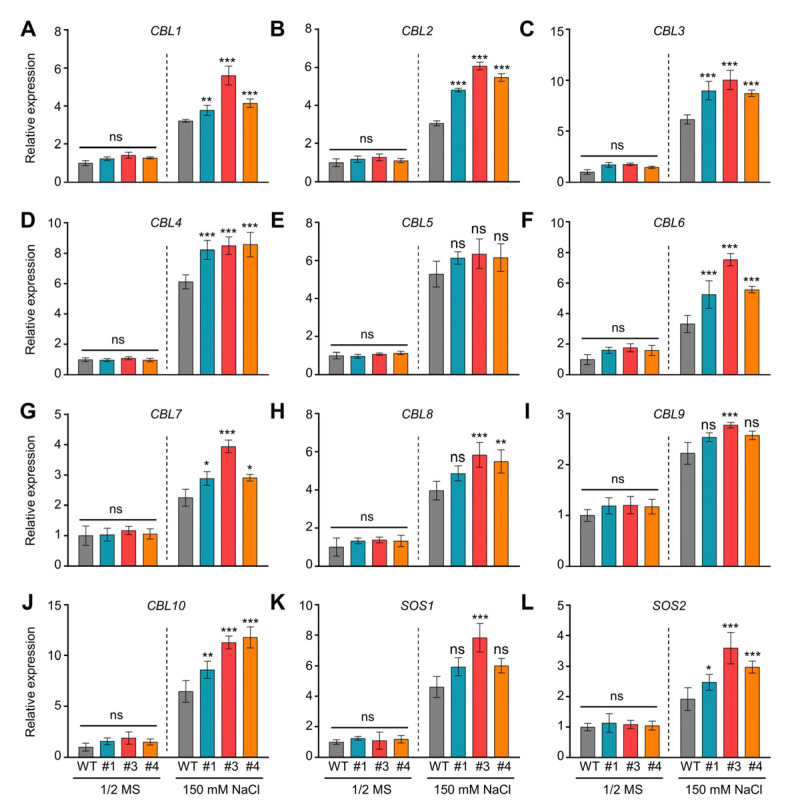
SiDi19-3 affects the expression of salt tolerance related genes in *Arabidopsis*. Expression levels of *CBL* (**A**–**J**) and *SOS* (**K**,**L**) genes in wild type (WT) and *SiDi19-3* transgenic *Arabidopsis* seedlings treated with 200 mM NaCl for 24 h or not. *AtGAPDH* and *AtUBQ10* were used as two normalizing expression values within the calculation leading to the relative expression values. Data shown are means of three biological replicates. Data represent the mean ± SEM of three biological repeats. Student’s *t*-test indicated the significance at * *p* < 0.05, ** *p* < 0.01, and *** *p* < 0.001 levels. ns, no significance.

To confirm its function, *SiDi19-3* was heterologously expressed in *Arabidopsis thaliana* Col-0 plants. Transgenic *Arabidopsis* lines #1, #3, and #4 indicated higher *SiDi19-3* transcript levels (Figure 4A). Under salt stress, the *SiDi19-3* transgenic *Arabidopsis* exhibited faster germination rate and growth with higher fresh weight and longer primary root length than those of the wild type (WT; Figure 4B–F). Furthermore, expression of *SiDi19-3* increased the expression of *AtCBL1-10* and *AtSOS1-2* but not *AtCBL5* in *Arabidopsis* seedlings under salt stress (Figure 5). Under normal conditions, no change was observed in germination rate, fresh weight, and primary root length nor in the expression of *NHXs*, *SOSs,* and *CBLs* in *Arabidopsis*. These results demonstrated that SiDi19-3 also acts as a positive regulator of salt stress in *Arabidopsis*.

### 2.5. Identification of SiDi19 Gene Family in Foxtail Millet

**Figure 6:**
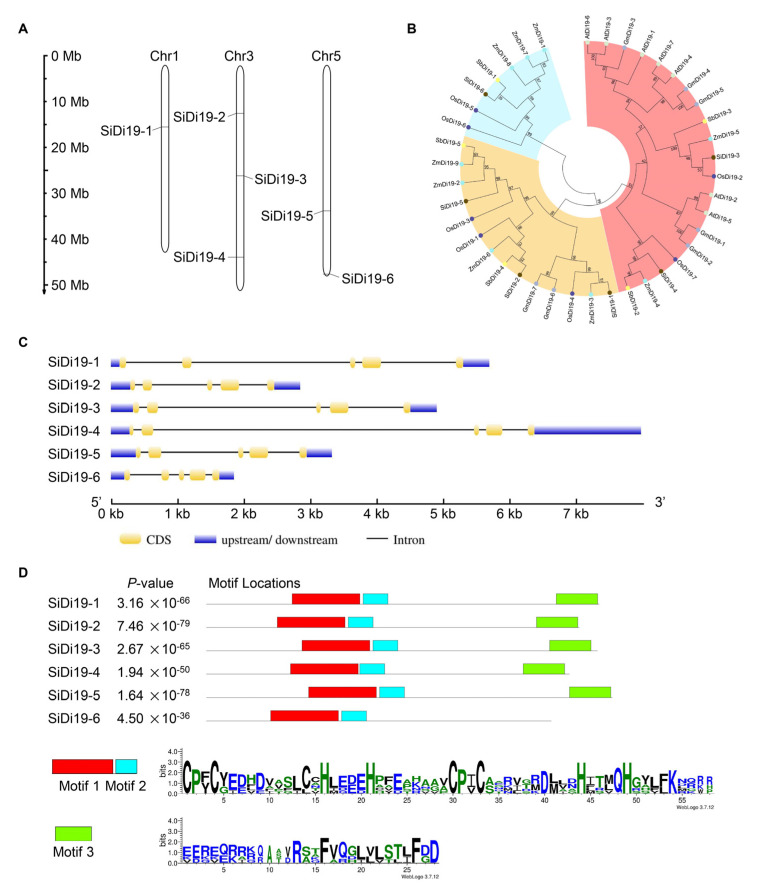
Analysis of *SiDi19* genes in foxtail millet. (**A**) Distribution of *SiDi19-3* genes on the chromosomes of foxtail millet. (**B**) The tree was reconstructed based on the highly conserved zf-Di19 domain of Di19 proteins in *Setaria italica*, *Oryza sativa*, *Arabidopsis*, *Sorghum bicolor*, and *Zea mays*. Bootstrap values from 1000 replicates are indicated at each node of the branches. (**C**) Gene structures of six *SiDi19* genes in foxtail millet created at GSDS website. (**D**) Conserved motif analysis according to multiple sequence alignments of SiDi19 proteins using the MEME website.

To determine whether other *SiDi19* members in foxtail millet exhibited a similar response to salt stress and other abiotic stressors, we searched the ‘Yugu1’ genome assembly containing Di19 sequences from foxtail millet (Supplemental Table S1). We found that *SiDi19* genes were unevenly distributed among nine chromosomes (chr) in foxtail millet. In comparison, chr3 contained three *SiDi19* genes, chr5 had two, and chr1 had only one *SiDi19* gene. Based on their chromosomal placement, the *SiDi19* genes were named *SiDi19-1*—*SiDi19-6* (Figure 6A). A bioinformatical neighbor-joining tree analysis divided *Di19* genes from *Setaria italica*, *Oryza sativa*, *Sorghum bicolor*, *Zea mays*, *Glycine max*, and *Arabidopsis* into three subfamilies that were similar to each other (Figure 6B), indicating that Di19 probably plays conserved roles among plant species. According to gene structural analysis, all *SiDi19* genes had the same structure, consisting of five variable-length exons and four introns (Figure 6C). It is interesting that all Di19 proteins have two conserved motifs except SiDi19-6, which indicate that they may have similar biological functions and that SiDi19-6 protein might play a distinct function in plants (Figure 6D).

### 2.6. Expression Patterns of SiDi19 Genes

**Figure 7:**
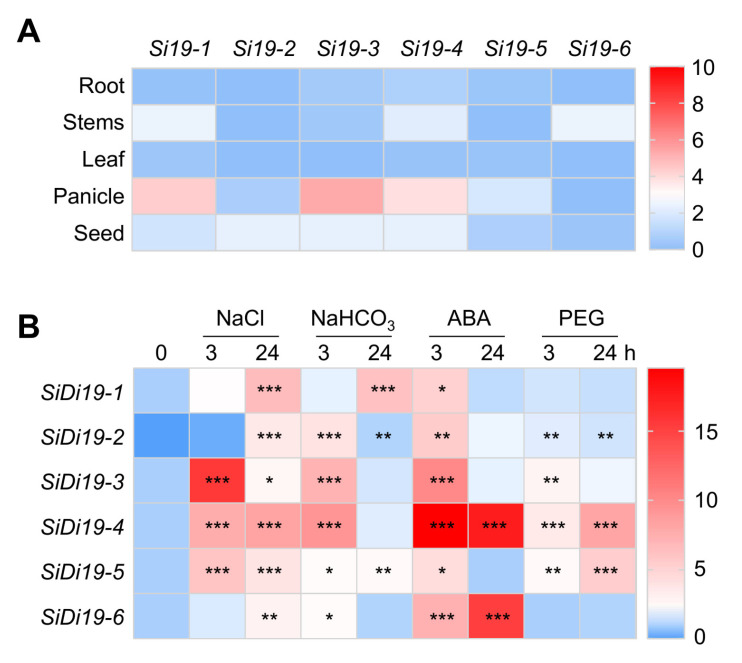
Expression patterns of *SiDi19* gene members in foxtail millet. (**A**) Quantitative reverse transcription polymerase chain reaction (RT-qPCR) analysis of *SiDi19* genes in roots, stems, leaves, panicles, and seeds. Root, stem, and leaf samples were collected from ten uniformly developed 14-day-old seedlings. Five approximately 5-cm-long, uniformly developed panicles from two-month-old Yugu1 seedlings were collected. Thirty mature seeds were collected. Each tissue sample was pooled and ground, and, respectively, three 100 mg tissue powder were weighed to extract RNA for RT-qPCR. All the tissues were collected three times from different plants for the three biological replicates. (**B**) RT-qPCR analysis of *SiDi19* genes in 7-day-old Yugu1 seedlings after treatment with 200 mM NaCl, 50 mM NaHCO_3_, 100 μΜ ABA, or 20% PEG6000 for 0, 3, and 24 h. *SiACTIN7* and *18S rRNA* were used as two normalizing expression values within the calculation leading to the relative expression values. Data shown are means of three biological replicates. Data represent the mean ± SEM of three biological repeats. Student′s *t*-test indicated the significance at * *p* < 0.05, ** *p* < 0.01, and *** *p* < 0.001 levels.

Next, using quantitative reverse transcription polymerase chain reaction (RT-qPCR) analysis, we examined the transcriptional levels of *SiDi19* genes in various organs. *SiDi19-1*, *SiDi19-4*, and *SiDi19-6* indicated higher transcript levels in stems than other members of the gene family, whereas *SiDi19-2*, *SiDi19-3*, and *SiDi19-4* were preferentially expressed in seeds (Figure 7A). Furthermore, *SiDi19-1*, *SiDi19-3*, and *SiDi19-4* indicated higher transcript levels in the panicles than other plant parts. All six *SiDi19* genes had low transcript levels in the roots and leaves. These gene expression patterns in different tissues suggested that some members may function redundantly at different developmental stages.

We initially examined the *cis*-elements in the promoters of *SiDi19* genes to determine whether any *SiDi19* genes other than *SiDi19-3* operated in response to abiotic stresses (Supplemental Table S2). Two or more ABA-responsive elements (ABREs) were predicted for all *SiDi19* promoters. An auxin-responsive element (TGA) was predicted in the promoters of *SiDi19-1*, *-3* and *-5.* Two or three ethylene response elements (ERFs) were predicted in the promoters of *SiDi19-2* and *-3.* More than one drought responsive element (DRE/MBS) was predicted in the promoters of *SiDi19-1*, *-2*, *-4*, and *-6.* One or two low-temperature responsive elements (LTRs) were predicted in the promoters of *SiDi19-2 to -*5. One to three salicylic acid-responsive elements (TCA/SARE) were predicted in the promoters of *SiDi19-1* and *-3*, respectively. More than one wound responsive element (WRE) was predicted in the promoters of all *SiDi19* genes except *SiDi19-2.* These results suggest the potential roles of *SiDi19* genes in hormone-mediated abiotic stress responses.

Furthermore, we detected the transcript levels of the *SiDi19* genes at 3 and 24 h after appropriate treatments. As depicted in Figure 7B, some treatments clearly induced the expression of several *SiDi19* genes. For example, all *SiDi19* genes were induced by NaCl, NaHCO_3_, and ABA treatments at 3 or 24 h, and all *SiDi19* genes, except *SiDi19-6,* were induced by PEG. Furthermore, *SiDi19-3* and -4 indicated the highest inducible levels in response to NaCl and ABA treatments. These results indicate the redundant roles of *SiDi19* genes in response to multiple ABA-mediated abiotic stresses in foxtail millet.

## 3. Discussion

Di19 members of several species control various abiotic stresses. Some constituents effectively controlled resistance to drought stress. For instance, AtDi19 improves drought tolerance in transgenic *Arabidopsis* [14]. In addition to AtDi19, OsDi19-4 [20], ZmDi19-1 [18], PheDi19-8 [21], and PtDi19-2/-7 [22] have similar functions. However, some Di19 adversely influence drought tolerance, such as TaDi19A [19], AtDi19-3 [29], and GmDi19-5 [16]. Additionally, ZmDi19-1 positively regulates salt tolerance [18], whereas AtDi19-3 [29], TaDi19A [19], GmDi19-5 [16], GhDi19-1, and GhDi19-2 [17] negatively regulate salt tolerance. Whether foxtail millet *Di19* genes function in response to abiotic stress is unknown. In this study, we discovered that SiDi19-3 favorably regulates the salt tolerance in foxtail millet and *Arabidopsis* (Figure 2 and Figure 4). Notably, both *SiDi19-3* and *SiDi19-4* displayed comparable expression patterns, including high transcript levels in the panicles and seeds, and exhibited similar responses to salt, alkaline, drought, and ABA treatments (Figure 7). These findings demonstrate that *Di19* genes play conserved roles in abiotic stress response across plant species. Furthermore, *SiDi19-3* and *SiDi19-4* probably control the development of panicles and seeds in foxtail millet; further studies are warranted to ascertain this since the involvement of *Di19* in plant development has not yet been reported. Further studies revealed that Di19 interacted with several proteins. Our previous report indicated that AtDi19-3 interacted with AtPLATZ2 [27]. Herein, we demonstrate that SiDi19-3 and SiPLATZ12 interacted with each other (Figure 1B–D). Both SiPLATZ12 and AtPLATZ2 exhibited detrimental roles in regulating plant salt stress [27,28]. However, SiDi19-3 positively regulates salt tolerance in foxtail millet and transgenic *Arabidopsis* (Figure 2 and Figure 4), whereas AtDi19-3 negatively controls salt tolerance in *Arabidopsis* [29]. In addition, AtDi19-3 interacts with AtIAA14, OsDi19-5, and OsIAA13 to control root elongation in *Arabidopsis* and rice [30]. Overexpression of GmDi19-5 increased sensitivity of transgenic *Arabidopsis* plants to salt, drought, oxidative, and ABA stresses and regulated expression of several ABA/stress-associated genes [16]. OsDi19-4, but not OsDi19-7, physically interacts with other Di19 members in yeast [31]. Members of Di19 appear to differently regulate abiotic stress through their different interacting proteins. The variant sequences between three conserved motifs may play roles in the interaction with different proteins (Figure 6D). However, precise regulatory framework is lacking.

The Di19 proteins belong to the Cys2His2 type transcription factor family, which regulates the expression of numerous genes involved in abiotic stress through the DNA-binding Cys2/His2 motifs [14]. For example, AtDi19-1 directly binds to the promoters of *PR1*, *PR2*, and *PR5* to regulate their expression in response to drought stress [14]. Similarly, AtDi19-3 positively controls the expression of *NIT1*, *ILL5*, *YUCCA*, *AUX1*, and *MYB77* [30]. By directly binding to the promoters of *OsASPG1* and *OsNAC18*, OsDi19-4 modulates the expression of ABA-responsive genes in rice [20]. In response to salt stress, ZmDi19-1 affects the expression of several stress-related genes, including *PR*, *RAB18*, *PDF,* and *COR15A* [18]. In contrast, the expression of *SOS2*, *ABI1/5*, *ABF3/4*, *RD29A/B*, *RD22,* and *DREB2A* is repressed by TaDi19A [19], AtDi19-3 [29], GmDi19-5 [16], and PheDi19-8 [21]. We found for the first time that SiDi19-3 localizes in the nucleus when expressed in hairy roots of foxtail millet (Figure 1A) and elevates the expression of the majority of the analyzed *SOS*, *CBL*, and *NHX* genes under salt stress (Figure 3 and Figure 5). Thus, SiDi19-3, similar to other Di19 members, controls the expression of many genes.

SiDi19-3 can control cytoplasmic Na^+^ homeostasis. *SOSs*, *CBLs*, and *NHXs* are important for cytoplasmic Na^+^ homeostasis. In *Arabidopsis*, SOS2-SOS3 (CBL4)/SCaBP8 (CBL10) complexes activate the transporter activities of *SOS1* and *NHXs* [5,32,33]. The results indicated that SiDi19-3 increased the expression of the majority of the analyzed *SOS*, *CBL*, and *NHX* genes (Figure 3 and Figure 5), indicating the involvement of SiDi19-3 in regulating cytoplasmic Na^+^ homeostasis. SiCBL4 and SiCIPK24 interact to attract SiCIPK24 to the plasma membrane. Compared with WT plants, *SiCIPK24* transgenic *Arabidopsis* plants are more resilient to salt stress [34]. *SiCBL5*-overexpressing foxtail millet plants demonstrate increased tolerance to salt stress. Furthermore, SiCBL5-SiCIPK24 affects SiSOS1 function in yeast cells [35]. We previously demonstrated that overexpression of *SiNHX2*, *SiCBL4,* and *SiCBL7* in the hairy roots of foxtail millet seedlings improved salt tolerance [28]. The salt tolerance of foxtail millet correlates with the expression of *SiNHX1* to *SiNHX4* [28]. Therefore, SiDi19-3 mediates salt tolerance by maintaining cytoplasmic Na^+^ homeostasis.

The development of foxtail millet might be influenced by SiDi19-3. Additionally, NHXs play roles in *Arabidopsis* stomatal control [36], plant growth [9,10,37], silique and seed development [38,39], and vacuolar K^+^ and pH homeostasis [9,10]. The up-regulation of *SOS*, *CBL*, and *NHX* gene expression by SiDi19-3 under salt stress (Figure 3 and Figure 5) indicates that SiDi19-3 may be involved in controlling foxtail millet growth and development under salt stress. The *SiDi19-3* transcript levels were higher in the panicles and in response to drought and ABA treatments (Figure 7), also indicating the possible participation of SiDi19-3 in foxtail millet panicle or seed development and drought response. These roles of SiDi19-3 and the underlying mechanisms merit additional studies.

## 4. Materials and Methods

### 4.1. Plant Materials and Growth Conditions

For investigation of gene expression and function, the foxtail millet (*S. italica*) cultivar ‘Yugu1’ and *Arabidopsis* Col-0 were employed. Foxtail millet seeds underwent sodium hypochlorite sterilization, were washed three times, and then were germinated for three days at 25 °C. Seven-day-old ‘Yugu1’ seedlings were exposed to Hoagland solution containing 200 mM NaCl, 50 mM NaHCO_3_, 100 μM ABA, or 20% (*w*/*v*) polyethylene glycol 6000 (PEG6000) for 3 and 24 h, respectively, for inducible expression analyses. The seedlings were collected and frozen in liquid nitrogen as soon as possible. Roots, stems, and leaves from 14-day-old seedlings, panicles from 2-month-old seedlings, and seeds of ‘Yugu1’ were collected and immediately frozen in liquid nitrogen for tissue expression analyses. The previously described techniques were used to grow foxtail millet seedlings with transgenic hairy roots [40].

The *A. thaliana* Col-0 was used as WT. The previously described techniques were used to grown *Arabidopsis* plants [41]. During the seed germination stage, seeds were exposed to 150 mM NaCl in 1/2 MS medium for 7 d. In a span of 7 d, the germinated seeds with 2 cm roots and 1 cm green shoots were counted daily. To induce salt stress, three-day-old seedlings germinated from seeds that were not salt treated of each genotype were transferred to 1/2 MS media containing 150 or 175 mM NaCl for an additional 14 d, and the root lengths and fresh weight of whole seedlings were measured. Three biological replicates of each experiment were performed.

### 4.2. Yeast Two-Hybrid (Y2H) Assays

The interaction between SiDi9-3 and SiPLATZ12 was tested using the Matchmaker GAL4 two-hybrid system (Clontech) as previously described [42]. Full-length coding sequences (CDSs) of SiDi9-3 and SiPLATZ12 were cloned into the pGBKT7 (BD) and pGADT7 (AD) vectors, respectively. The yeast strain AH109 was co-transformed with those constructs. Leu–Trp–His–Ade (QDO) was used for interaction selection, while double dropout (DDO) medium supplemented without Leu or Trp was used to grow the transformed cells. Primers used in this experiment are listed in Supplemental Table S3.

### 4.3. Bimolecular Fluorescence Complementation (BiFC) Assays

For these assays, the coding regions of SiDi19-3 and SiPLATZ12 were amplified and cloned into pSPYNE-35S (NYFP) and pSPYCE-35S (CYFP) vectors to fuse them with the N- or C-terminal YFP, respectively. The resultant *35S::*SiDi19-3-NYFP and *35S::*SiPLATZ12-CYFP constructs were transformed into *Agrobacterium tumefaciens* GV3101. The *35S::*P19 vector was co-transformed into three-week-old *N. benthamiana* leaves with *35S::*SiDi19-3-NYFP and *35S::*SiPLATZ12-CYFP, *35S::*SiDi19-3-NYFP and CYFP, NYFP, *35S::*SiPLATZ12-CYFP, NYFP, and CYFP. After 28 h of culture, the transformed tobacco leaves were monitored for reconstituted YFP fluorescence using an LSM880 confocal microscopy (Zeiss, Germany). The primers used in this experiment are listed in Supplemental Table S3.

### 4.4. Pull-Down Assays

For these assays, the CDSs of *SiDi19-3* and *SiPLATZ12* were cloned into pGEX-4T-1 and pCold-TF vectors to generate SiDi19-3-GST and SiPLATZ12-His constructs, respectively, as previously described [41]. Subsequently, the constructs were transformed into competent Rosetta *E. coli* cells. SiDi19-3-GST and SiPLATZ12-His proteins were induced by IPTG and were then used for protein purification using the His-Tagged Protein Purification Kit (CWBIO, Beijing, China) and BeyoGold GST-tag Purification Resin (BeyoGold, Shanghai, China). Next, SiDi19-3-GST or GST proteins were incubated with SiPLATZ12-His protein in 300 μL of binding buffer (P2262, Beyotime, Shanghai, China) for 1 h at 4 °C in a continuously rotator. The proteins were detected using immunoblotting with anti-GST or anti-His antibodies after elution from the beads. Signals were detected using a Chemiluminescence Imaging System (K4000, KCRX Biotechnology, Beijing, China). The primers used in this experiment are listed in Supplemental Table S3.

### 4.5. Subcellular Localization Analysis

The transgenic hairy-roots harboring pSiDi19-3:SiDi19-3-GFP were obtained using the previously described techniques [39] to analyze the subcellular location of SiDi19-3. LSM880 high-resolution laser confocal microscope (Zeiss, Germany) was utilized to view GFP fluorescence in the root tips of the transgenic hairy roots. The DAPI was used to detect the nuclear dye.

### 4.6. Generation of Transgenic Foxtail Millet and Arabidopsis

Under the control of the 35S promoter, the full-length CDS of *SiDi19-3* was amplified and cloned into PROKII vector. Based on an earlier study [39], the recombinant plasmid was transformed into *A. tumefaciens* K599. Positive transformants were inoculated into 20 mL of liquid solution and cultured with continuous shaking for 12 h. The culture was then centrifuged at 6000× *g* for 5 min. Subsequently, the cells were resuspended in 1/2 MS liquid medium to a final concentration with OD_600_ = 1.0. Three-day-old shoot tips were cut and incubated with *A. tumefaciens* containing *35S::*SiDi19-3 or the empty vector at 28 °C for 10–20 min with continuous shaking. The transformed shoot tips were transferred onto 1/2 MS solid medium supplemented with 100 mg/L timentin overnight to induce root formation [39]. Approximately 7 d later, transgenic hairy roots were confirmed using RT-qPCR. Twenty transgenic seedlings were transferred into 1/2 MS liquid medium with or without 150 mM NaCl for an additional 7 d. The phenotype was photographed, and root length was measured. Three biological replicates of each experiment were performed.

Transgenic *Arabidopsis* containing *35S::*SiDi19-3 was simultaneously generated using the floral-dipping method as previously described [43]. T_3_ transgenic *Arabidopsis* plants were selected using 1/2 MS medium supplemented with 50 mg/L kanamycin and confirmed by RT-qPCR using the primers listed in Supplemental Table S3.

### 4.7. Identification of SiDi19 Genes in Foxtail Millet

Di19 family members from *Arabidopsis* were used to BLAST the foxtail millet genome in Phytozome V12 (https://phytozome.jgi.doe.gov (accessed on 19 April 2022)) to identify the SiDi19 genes. The isoelectric point and molecular weight were predicted using ExPASy Proteomics Server (http://expasy.org/ (accessed on 19 April 2022)). An evolutionary tree was generated using MEGA6 software and a phylogenetic evolutionary tree was constructed using neighbor-joining analysis and bootstrap method, with 1000 replicates. *Di19* genes from *Oryza sativa*, *Arabidopsis*, *Sorghum bicolor*, *Zea mays*, and *Glycine max* were previously identified [13,16,29]. The gene structures of SiDi19 members were analyzed using the Gene Structure Display Server (GSDS; http://gsds.cbi.pku.edu.cn/ (accessed on 19 April 2022)). MEME (https://meme-suite.org/meme/tools/meme (accessed on 19 April 2022)) was used to analyze the conserved motifs. The *cis*-elements in the promoters of the SiDi19 genes were predicted using PlantCARE (http://bioinformatics.psb.ugent.be/webtools/plantcare/html/ (accessed on 19 April 2022)).

### 4.8. RNA Extraction and RT-qPCR

Total RNAs was extracted from the indicated tissues and seedlings treated with the indicated stimuli using RNAiso Plus (Takara, Ohtsu, Japan). Two micrograms of total RNAs were used to synthesize cDNAs using the PrimeScriptTM RT reagent kit (TaKaRa, Ohtsu, Japan) according to the manufacturer’s instructions. Further, RT-qPCR was performed using ChamQ Universal SYBR qPCR Master Mix (Vazyme, Biotech Co., Ltd.) in a three-step program on a CFX96TM Real-Time PCR Detection System (Bio-Rad, Hercules, CA, USA). *SiACTIN7* and 18S rRNA were used as two normalizing expression values within the calculation leading to the relative expression values in foxtail millet [40,44]. *AtGAPDH* and *AtUBQ10* were used as two normalizing expression values within the calculation leading to the relative expression values in *Arabidopsis* [41,42]. Three biological replicates were performed. The Primers used are listed in Supplementary Table S3.

## Data Availability

The data and materials supporting the findings of this study are available from the corresponding author upon request.

## Supplementary Materials

The following supporting information can be downloaded at: https://www.mdpi.com/article/10.3390/ijms24032592/s1.
