# Supplementary material for: Transcription Factor SiDi19-3 Enhances Salt Tolerance of Foxtail Millet and Arabidopsis
Source: Int J Mol Sci. 2023 Jan 30;24(3):2592. doi: 10.3390/ijms24032592 (PMC9917086; doi:10.3390/ijms24032592)
Supplement: Supplementary file 1 [file ijms-24-02592-s001.zip › Table S1. Identification of Di19 genes from foxtail millet.pdf]

Supplemental Table S1： Identification of *Di19* genes from foxtail millet

Gene Name	Gene ID	Chr.	Number of amino acids	Molecular weight	*PI*
SiDi19-1	Seita.1G137300.1	1	237aa	26831.81	6.49
SiDi19-2	Seita.3G147600.1	3	225aa	25180.17	5.56
SiDi19-3	Seita.3G274200.1	3	236aa	25737.64	5.23
SiDi19-4	Seita.3G337800.1	3	219aa	24256.09	4.51
SiDi19-5	Seita.5G266000.1	5	245aa	27310.45	4.95
SiDi19-6	Seita.5G467500.1	5	208aa	23657.62	5.58
