# Supplementary material for: Transcription Factor SiDi19-3 Enhances Salt Tolerance of Foxtail Millet and Arabidopsis
Source: Int J Mol Sci. 2023 Jan 30;24(3):2592. doi: 10.3390/ijms24032592 (PMC9917086; doi:10.3390/ijms24032592)
Supplement: Supplementary file 1 [file ijms-24-02592-s001.zip › Table S2. Analysis of various response elements in promoter regions..pdf]

Supplemental Table S2: Analysis of various response elements in promoter regions.

Name	ABRE	TGA	ERE	MBS/DRE	GT1/SRE	LTR	HSE	TCA/SARE	JARE	WRE
Di19-1	4	1	0	1	0	0	0	3	0	4
Di19-2	8	1	3	6	0	1	0	0	0	0
Di19-3	4	1	2	0	0	1	0	1	0	2
Di19-4	3	0	0	1	0	2	0	0	0	1
Di19-5	2	1	0	0	0	2	0	0	0	2
Di19-6	2	0	0	3	1	0	0	0	0	1
